# The Natural-Based Antitumor Compound T21 Decreases Survivin Levels through Potent STAT3 Inhibition in Lung Cancer Models

**DOI:** 10.3390/biom9080361

**Published:** 2019-08-13

**Authors:** David Martínez-García, Marta Pérez-Hernández, Luís Korrodi-Gregório, Roberto Quesada, Ricard Ramos, Núria Baixeras, Ricardo Pérez-Tomás, Vanessa Soto-Cerrato

**Affiliations:** 1 Department of Pathology and Experimental Therapeutics, Faculty of Medicine and Health Sciences, Universitat de Barcelona, 08905 Barcelona, Spain; 2 Oncobell Program, Institut d’Investigació Biomèdica de Bellvitge (IDIBELL), L’Hospitalet de Llobregat, 08908 Barcelona, Spain; 3 Department of Chemistry, Universidad de Burgos, 09001 Burgos, Spain; 4 Department of Thoracic Surgery and University of Barcelona, Hospital Universitari de Bellvitge, L’Hospitalet de Llobregat, 08907 Barcelona, Spain; 5 Department of Pathology, Hospital Universitari de Bellvitge-IDIBELL, L’Hospitalet de Llobregat, 08907 Barcelona, Spain

**Keywords:** natural-based compound, anticancer therapy, lung cancer, survivin, apoptosis, STAT3

## Abstract

Lung cancer is the leading cause of cancer-related deaths worldwide; hence novel treatments for this malignancy are eagerly needed. Since natural-based compounds represent a rich source of novel chemical entities in drug discovery, we have focused our attention on tambjamines, natural compounds isolated from marine invertebrates that have shown diverse pharmacological activities. Based on these structures, we have recently identified the novel indole-based tambjamine analog 21 (T21) as a promising antitumor agent, which modulates the expression of apoptotic proteins such as survivin. This antiapoptotic protein plays an important role in carcinogenesis and chemoresistance. In this work, we have elucidated the molecular mechanism by which the anticancer compound T21 exerts survivin inhibition and have validated this protein as a therapeutic target in different lung cancer models. T21 was able to reduce survivin protein levels in vitro by repressing its gene expression through the blockade of Janus kinase/Signal Transducer and Activator of Transcription-3 (JAK/STAT3)/survivin signaling pathway. Interestingly, this occurred even when the pathway was overstimulated with its ligand interleukin 6 (IL-6), which is frequently overexpressed in lung cancer patients who show poor clinical outcomes. Altogether, these results show T21 as a potent anticancer compound that effectively decreases survivin levels through STAT3 inhibition in lung cancer, appearing as a promising therapeutic drug for cancer treatment.

## 1. Introduction

Lung cancer is the leading cause of cancer-related deaths in both men and women worldwide, accounting for more than 2.1 million new cases and more than 1.8 million deaths estimated in 2018 [1]. This malignancy is broadly categorized into small cell lung cancer (SCLC) and non-small cell lung cancer (NSCLC), which represents the 85% of all lung cancers diagnosed. NSCLC is further divided in three major subtypes based on their histology: adenocarcinoma, squamous cell lung carcinoma (SQCLC) and large cell carcinoma, corresponding to 45–50%, 25–30% and 5–10% of all diagnosed NSCLC, respectively [2]. The current standard of care for lung cancer differs according to the tumor histological type, the stage of cancer, possible side effects, and overall patient health. Considering this, surgical resection, radiotherapy, conventional platinum-based doublet chemotherapy (cisplatin generally in combination with pemetrexed/Alimpta^®^ or gemcitabine), immunotherapy (anti PD-1, mostly nivolumab/Opdivo^®^)), and targeted therapy are the main options to treat lung cancer [3]. Nevertheless, despite all the available therapeutic options, the five-year survival rate of lung cancer is low, 18.6%, according to the National Cancer Institute (NCI). Therefore, novel therapeutic strategies should be developed to increase the therapeutic options available for the treatment of this malignancy.

Natural-based compounds constitute an important research area for cancer drug discovery, with numerous compounds showing therapeutic potential in most cancer types. Interestingly, over 70% of anticancer compounds in clinical use derive from natural products, such as the marine organism-derived compounds cytarabine (Cytosar), travectedin (Yondelis), eribulin mesylate (Halaven) and the conjugated antibody brentuximab vedotin (Acentris) [4]. In particular, marine organisms are gaining interest for providing a huge array of biologically active metabolites for the development of new anticancer agents. The natural alkaloids tambjamines, originally isolated from marine invertebrates, have shown a wide spectrum of pharmacological properties [5]. In this regard, we have demonstrated that the indole-based tambjamine analog 21 (T21) exerts a potent anticancer effect in vitro and a significant therapeutic effect, with a favorable safety profile, in vivo in lung cancer mice models [6]. Moreover, T21 was able to modulate apoptotic protein levels, including survivin. As a member of the inhibitor of apoptosis (IAP) family, survivin plays an important role in tumorigenesis, metastasis and therapy resistance by promoting cell division and inhibiting apoptosis [7]. Furthermore, survivin is overexpressed in cancer cells, while in most normal finally differentiated tissues is almost undetectable [8]. Altogether, these features advocate survivin as an ideal therapeutic target to treat cancer and hence, T21 may be a promising future chemotherapeutic agent. In fact, several molecular approaches that block survivin expression and/or function are emerging as promising therapeutic strategies in cancer by sensitizing tumor cells to apoptosis, minimally affecting non-tumor cells [9].

Therefore, in this work we have deeply analyzed, for the first time, the molecular mechanism of action by which a recently described natural-derived compound called T21 inhibits survivin, inducing its anticancer effects in vitro as well as in in vivo mice models, validating survivin as a promising therapeutic target for lung cancer treatment.

## 2. Materials and Methods

### 2.1. Human Samples

Fresh squamous cell lung carcinoma tissue and adjacent non-tumor lung tissue samples were obtained from patients during resection surgery at Bellvitge University Hospital in Barcelona, Spain. The study was conducted in accordance with the Declaration of Helsinki ethical guidelines and informed consent was obtained from all patients included in the study. All study protocols were approved by the Clinical Research Ethics Board of Bellvitge University Hospital and by the local Ethics Committee (PR003/13). The histological typing was confirmed by the Pathology Department at the aforementioned Hospital. All the human tissue samples were preserved in RNAlater™ (Cat#76104, Qiagen) and stored at liquid nitrogen before being processed.

### 2.2. Reagents

Tambjamine-21 analogue (T21) was synthesized as previously reported [6], dissolved at 10 mmol/L in dimethyl sulfoxide (DMSO) and stored at −20 °C. Cycloheximide (CHX; Cat#C7698-IG) from Sigma-Aldrich was dissolved in ethanol at a stock solution of 100 mg/mL and stored at −20 °C. Interleukin 6 (IL-6; Cat#IL006) was purchased from EMD Millipore and dissolved in 1× phosphate-buffered saline (PBS; Cat#02-020, Biological Industries, Beit Haemek, Israel) with calcium and magnesium supplemented with 0.1% bovine serum albumin (BSA; Cat#A7906, Sigma-Aldrich, St Louis MO, USA) at a stock solution of 100 µg/mL and stored at −80 °C. Hoechst 33,342 (Cat# B2261) was purchased from Sigma-Aldrich.

### 2.3. Antibodies

The antibodies used in this study were obtained from the following sources: anti-survivin (71G4B7, Cat#2808), anti-XIAP (3B6, Cat#2045), anti-phospho-JAK1 (Y1034/1035; Cat#3331), anti-phospho-STAT3 (Y705; D3A7, Cat#9145), anti-cleaved PARP (Cat#5625T), anti-cleaved caspase 3 (Cat#9664) and anti-phospho-JAK2 (Y1007/1008; Cat#3771) from Cell Signaling Technology Inc. (Beverly, MA, USA); anti-actin (I-19, Cat#sc-1616), anti-GAPDH (0411, Cat#sc-47724), anti-JAK1 (B-3, Cat#376996), anti-phospho-STAT3 (Y705; B-7, Cat#sc-8059), anti-STAT3 (F-2, Cat#sc-8019), from Santa Cruz Biotechnology Inc. (Santa Cruz, CA, USA); anti-vinculin (Cat#V-4505) from Sigma-Aldrich. Antibody binding was detected with donkey anti-mouse IgG-HRP (Cat#A16017), donkey anti-rabbit IgG-HRP (Cat#A16029) and donkey anti-goat IgG-HRP (Cat#A15999) from Thermo Fisher Scientific Inc. (Waltham, MA, USA); Alexa Fluor™ 488-conjugated donkey anti-mouse (Cat#A31572, Molecular Probes, Eugene, OR, USA) was used for antibody binding detection in immunofluorescence assays.

### 2.4. Cell Lines and Culture Conditions

Human cell lines SW900 and H520 (squamous lung carcinoma), A549 (lung adenocarcinoma), DMS53 (small cell lung carcinoma) and HFL-1 (lung fibroblasts), were obtained from the American Type Culture Collection (ATCC). SQCLC, adenocarcinoma and lung fibroblasts cells were cultured (passage number 10–25) in Roswell Park Memorial Institute medium (RPMI, Cat# 01-104), Dulbecco’s modified Eagle’s medium (DMEM, Cat#01-055) and Ham’s F-12 (Cat#01-095) (Biological Industries), respectively. All of them were supplemented with 10% heat-inactivated fetal bovine serum (FBS Gibco™; Cat#10270106, Life Technologies), 100 units/mL penicillin, 100 μg/mL streptomycin, and 2 mM L-glutamine (all from Biological Industries). Non-essential amino acids (NEAA; Cat#X0557, Biowest; 1:100) were also used for HFL-1 culture (Biological Industries). 15 mM of HEPES buffer solution (Cat#03-025, Biological Industries) was also used for H520 culture. Cells were grown at 37 °C in a humidified incubator (Thermo Fisher Scientific Inc.) with 5% CO_2_ atmosphere. The cells were mycoplasma tested using a standard PCR technique after thawing.

### 2.5. Gene Expression Analysis

Gene expression levels of BIRC5 were evaluated by Reverse Transcription quantitative-PCR (RT-qPCR) analysis. Total RNA was isolated and purified from 30 mg of frozen tissue samples using the column-based RNeasy Mini Kit (Qiagen) and following the manufacturer’s standard protocol. Total RNA concentration and purity were checked in a nano spectrophotometer (Implen GmbH, Munchen, Germany) and integrity was analyzed using an Agilent 2100 Bioanalyzer (Agilent Technologies, Santa Clara, CA, USA). Samples with higher RNA integrity number (RIN) were selected (7 lung cancer samples and their paired normal tissue samples from the same patient). RNA amounts from selected tissue samples were equally pooled to create a sample (Σnon-tumoral and Σtumoral) and their concentration, purity and integrity were re-checked. For the reverse transcription, 1 μg of total RNA was used for cDNA synthesis using a mixture of random hexamers and oligo-dT primers and following the RT2 First Strand Kit protocol (Qiagen). Then, reverse transcription was confirmed through actin beta (ACTB) gene amplification by standard PCR procedure (BIOTAQ DNA Polymerase; BIOLINE). For survivin expression analysis in A549 and SW900 cells, 1.25 × 10^5^ cells/mL were seeded and after 24 h they were treated in absence or presence of T21 for 6 or 16 h (IC_50_ concentrations). RNA was purified and cDNA obtained as described above. Specific oligonucleotide primers and probes for BIRC5 (Hs00153353_m1), and ACTB (Hs99999903_m1), were purchased as Assay-on-Demand Gene Expression Products (Applied Biosystems). TaqMan PCR reactions were performed on cDNA samples using TaqMan Universal PCR Master Mix (Applied Biosystems, Fosters city, CA, USA) and ABI PRISM 7900 HT Fast Real-Time PCR system (Applied Biosystems). Gene expression levels were quantified and normalized using ACTB as a house keeping gene and relative mRNA expression was calculated in relation to the healthy samples. Ct values were determined using ExpressionSuite software (version 1.0.3, Applied Biosystems) and are presented as mean ± SD of three independent experiments.

### 2.6. Cell Viability Assays

Cell viability was evaluated using the methylthiazoletetrazolium (MTT, Sigma-Aldrich, Merck KGaA) colorimetric assay. Cells were harvested (10^5^ cells/mL) in 96-well plates and allowed to grow overnight. At the following day, T21 was added to the cells at different ranging concentrations (0.8–100 μmol/L) or vehicle solution (DMSO, Sigma-Aldrich, Merck KGaA) to control cells. Cells were incubated for 24 h and after the treatment period, 10 μL of MTT (5 mg/mL) were added and the plates were incubated for 2 h at 37 °C. Crystals were dissolved in 100 mL of DMSO and reading was done in a spectrophotometer at 570 nm using a multiwell plate reader (Multiskan FC, Thermo Fisher Scientific Inc.). Cell viability and inhibitory concentration (IC) values were obtained using GraphPad Prism V5.0 for Windows (GraphPad Software). All data are shown as the mean value ± SD of three independent experiments.

### 2.7. Clonogenic Assay

A549 cells at 10^5^ cells/mL were seeded (1 mL) in 24-well plate and incubated overnight to allow attachment. Cells were treated for 24 h with T21 at 2.5–10 µM and the same percentage of DMSO was added to the control cells. Then, cells were counted and 200 viable cells were seeded in a final volume of 3 mL in a 6-well plate and allowed to growth for 1 week. Medium was removed and cell colonies were fixed coloured with a mixture of glutaraldehyde (6% *v/v*) and crystal violet (0.5% *w/v*) for 20 min at room temperature and were counted.

### 2.8. Western Blot Analysis

For the evaluation of the molecular effects after 48 h of survivin silencing, A549 and H520 cells were seeded in 6-wells plates at a density of 1.25 × 10^5^ cells/mL in a volume of 2 mL of medium without antibiotics. The day after, cells at 70–90% of confluence were transfected with 250 pmol of small interfering RNA (siRNA) against survivin (Cat#4390824; Thermo Fisher Scientific Inc.) or scrambled siRNA (Cat#4390843; Thermo Fisher Scientific Inc.) using Lipofectamine^®^ 2000 reagent (Thermo Fisher Scientific Inc.) and following manufacturer’s standard protocol.

For the study of T21 cellular effects, A549, SW900, H520 and DMS53 were seeded in 100-mm cell culture plates (1.25 × 10^5^ cells/mL) and allowed to grow for 24 h. Then they were treated with different inhibitory concentrations (IC) of T21 compound (IC_25_, IC_50_ and IC_75_ values in µM) for 24 h or the IC_50_ value during different time periods (4, 8, 16, 24 h).

For protein synthesis inhibition, A549 and SW900 were seeded in a 6-well plate (1.25 × 10^5^ cells/mL) and allowed to grow for 24 h. Then they were treated with CHX at 100 µg/mL for 30 min followed by T21 treatment at IC_50_ during 24 h.

For the stimulation of STAT3 pathway with IL-6, A549 cells were seeded in 60-mm culture plates (1.25 × 10^5^ cells/mL) for 24 h, and then were starved in serum-free medium for at least another 12 h. Next, cells were stimulated with IL-6 at different concentrations (1, 5 and 10 ng/mL) for 30 min. Similarly, after starvation, A549 cells were pretreated with IC_50_ T21 for 4 h followed by 5 ng/mL of IL-6 stimulation for 30 min.

In all experiments, whole cell lysates, from the selected tissue samples or from cultured cells, were prepared with ice cold lysis buffer containing 0.1% SDS, 1% NP-40, 0.5% sodium deoxycholate, 50 mmol/L sodium fluoride, 40 mmol/L β-glycerophosphate, 200 µmol/L sodium orthovanadate, 1 mmol/L phenylmethylsulfonyl fluoride (all from Sigma-Aldrich), and protease inhibitor cocktail (Cat#11836170001, Roche Diagnostics) in 1× PBS followed by its homogenization, using a tissue grinder (Cat#431-0100, VWR International) in case of the tissue samples. Protein concentration was determined by BCA protein assay (Cat#23225, Pierce™, Thermo Fisher Scientific Inc.) using BSA protein (Sigma-Aldrich) as a standard. For western blot analysis, 40–50 µg of protein extract were first separated by SDS-PAGE and transferred to Immobilon-P polyvinylidene difluoride (PVDF) membranes (EMD Millipore, Merck KGaG). Membranes were blocked in either 5% non-fat dry milk or BSA, both diluted in Tris-buffered saline (TBS)-Tween (50 mmol/L Tris-HCl pH 7.5, 150 mmol/L NaCl, 0.1% Tween-20) for 1 h and then incubated overnight with primary antibodies, according to the manufacturer’s instructions. Actin, vinculin or GAPDH (Glyceraldehyde 3-phosphate dehydrogenase) were used as gel loading controls. The results shown are representative of Western blot data analysis obtained from at least three independent experiments. Images were captured on an Image Quant LAS 500 (GE Healthcare) using ECL™ Western blotting detection reagent (Cat#RPN2106, Amersham, GE Healthcare) and band densitometries were retrieved using the Image Studio Lite software (v5.2, LI-COR Biosciences).

### 2.9. Immunofluorescence Staining

HFL-1, A549 and SW900 cells (1.25 × 10^5^ cells/mL) were seeded in a 12-well plate containing FBS-coated glass coverslips for 24 h. For the IL-6 experiment, A549 cells (1.25 × 10^5^ cells/mL) were seeded in a 12-well plate containing FBS-coated glass coverslips for 24 h and then were starved in serum-free medium for at least another 12 h. Then, cells were pretreated with IC_50_ T21 for 4 h followed by 5 ng/mL of IL-6 stimulation for 30 min. Next, all cells were washed twice with 1× PBS and fixed with 4% paraformaldehyde for 20 min. Fixed cells were permeabilized by 0.2% Triton X-100 and then blocked with 1% BSA in 1× PBS for 1 h. Cells were incubated overnight at 4 °C with anti-survivin antibody at a dilution of 1:500 or anti-phospho-STAT3 (Cell Signaling) at a dilution of 1:100. Cells were then washed with 1× PBS and incubated with Alexa Fluor™ 488-conjugated goat anti-rabbit (Molecular Probes) at 1:400 dilution for 1 h at room temperature. At the same time, the cell nuclei were stained with 2 µg/mL hoechst 33,342 (Cat# B2261, Sigma-Aldrich). Afterwards, coverslips were washed with 1× PBS and were placed on the slides using Mowiol™ (Sigma-Aldrich). The immunofluorescence images were acquired using a Carl Zeiss LSM 880 spectral confocal laser scanning microscope (Carl Zeiss Microscopy GmbH, Jena, Germany) equipped with a multiline argon laser (458 nm, 488 nm and 514 nm), 405nm and 561nm diode lasers and 633 nm He/Ne laser (Centres Científics i Tecnològics, Universitat de Barcelona, Bellvitge Campus, Barcelona, Spain) using a 63× oil immersion objective (1.4 numerical aperture) an image resolution of 1024 × 1024 pixels. Representative images from three independent experiments are shown.

### 2.10. Immunohistochemistry Analysis

For in vivo studies, five-week-old female Crl:NU-Foxn1nu mice strain (Envigo) were used to generate a subcutaneous xenograft model. All animal studies were approved by the Autonomic Ethic Committee (Generalitat de Catalunya) under the protocol 9111. DMS53 cells (4.5 × 10^6^ cells) suspended in a 1:1 solution of RPMI1460:Matrigel (BD Bioscience) were implanted subcutaneously in the flank of mice. Mice bearing homogenous subcutaneous tumors (approximately 150–200 mm^3^) were randomly allocated to two treatment groups (*n* = 7/treatment) and intraperitoneally administrated with T21 (diluted in 7.5% DMSO/0.8% Tween-80) at a dose of 6 mg/kg in alternated days during 20 days. After the final dose of the treatment, animals were sacrificed, tumors dissected out and embedded in paraffin for immunohistochemistry staining as follows: 4 µm sections were cut, deparaffinized and after antigen retrieval in 10 mmol/L sodium citrate buffer with 0.05% Tween-20 in the microwave at sub-boiling temperature (95–98 °C) for 20 min, slides were washed 2 times with distilled H_2_O (dH_2_O) of 5 min each. Endogen peroxidase was blocked by incubation in 3% H_2_O_2_ for 5 min at room temperature following by washing steps for 5 min twice, with dH_2_O and PBS. Slides were blocked with normal goat serum in a 1:30 dilution for 1 h at room temperature and incubated with anti-survivin antibody diluted 1:400 in PBS overnight at 4 °C in a wet chamber. Afterwards, slides were washed 3 times in PBS for 5 min each and incubated with secondary antibody coupled with HRP at 1:100 dilution in PBS for 1 h, at room temperature. Then, slides were washed 3 times with PBS 0.1% Tween-20 for 5 min each and signal was developed by incubation with DAB (3,3′-diaminobenzidine) (Cat#D8001, Sigma) for 10 min at room temperature. Finally, slides were washed for 5 min with dH_2_O, counterstain with Hematoxylin (Cat#A3865, PanReac AppliChem, Barcelona, Spain), dehydrated, and mounted with DPX (Cat#100579, Merck, Madrid, Spain). Samples were observed in a Nikon Eclipse E800 microscope and images were taken with the camera ProgRes CFscan.

### 2.11. Statistical and Data Mining Analyses

For statistical analysis of single point qPCR results and western blot data, t-Student test and one-way ANOVA with post hoc Tukey analysis, were carried out using the Statgraphics plus 5.1 statistical Software, respectively. Statistically significant differences, *p* < 0.05, *p* < 0.01 and *p* < 0.001, are represented by *, ** and ***, respectively.

## 3. Results

### 3.1. Survivin Validation as A Promising Therapeutic Target in All Lung Cancer Subtypes

**Figure 1:**
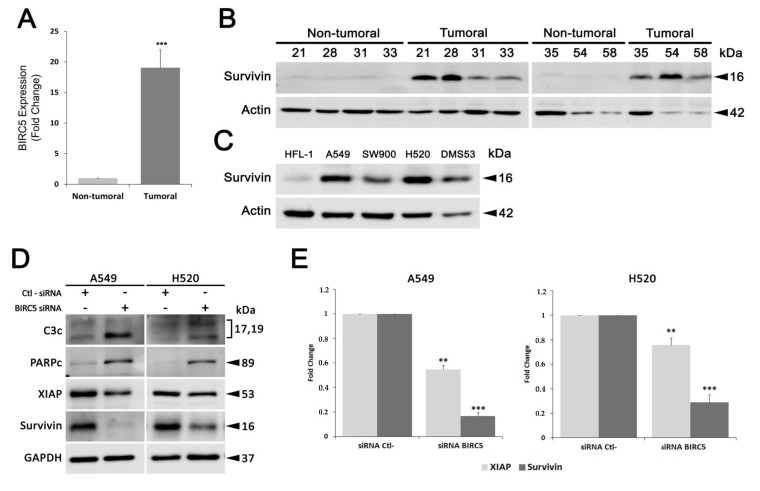
Survivin expression levels in lung cancer tumor samples and survivin functional validation in cellular models. (**A**) qPCR validation of BIRC5 expression levels. Fold changes of gene expression were calculated using β-actin as the housekeeping gene. (**B**,**C**), survivin levels were analyzed using whole cell lysates from patient tissue samples and lung cell lines by Western blot analysis. (**D**,**E**), After 48 h of transfection with siRNA against survivin mRNA, the expression of survivin, XIAP and pro-apoptotic proteins was analyzed by Western blot analysis in A549 and H520 cell lines. Protein levels were normalized with their respective loading controls in each blot. Results were obtained from at least three independent experiments. Bars represent the mean ± SD. Statistically significant results are indicated as *, *p*-value < 0.05; **, *p*-value < 0.01 and ***, *p*-value < 0.001. C3c, cleaved caspase 3; PARPc, cleaved PARP.

Since the anticancer T21 compound potently decreases the protein levels of the antiapoptotic protein called survivin, expression levels of this potential therapeutic target were evaluated in tumor samples from SQCLC patients through both specific qPCR analysis and survivin protein immunodetection (Figure 1A,B). In accordance with other tumors, BIRC5 was confirmed to be up-regulated more than 19 times in SQCLC samples using qPCR and consequently, survivin expression in tumor samples was much higher than in non-tumor samples. Furthermore, we wanted to analyze whether this protein may also be a therapeutic target for all the most prevalent lung cancer histological subtypes. Hence, we analyzed survivin overexpression in the adenocarcinoma cell line A549, the small cell lung cancer cell line DMS53 as well as in the SQCLC cell lines, SW900 and H520. All four tumor cell lines overexpress survivin compared to non-tumor human lung fibroblasts HFL-1 (Figure 1C; Figure S1). Interestingly, survivin has a dual role in cancer, both promoting cell cycle progression and inhibiting apoptosis [8]. Therefore, in order to validate survivin as an effective therapeutic target in lung cancer, we proposed to perform a loss-of-function assay by inhibiting survivin expression. In this context, A549 and H520 cells were transfected with a siRNA against survivin mRNA or a control siRNA designed to have no specific target in the cell. After 48 h of transfection, there was no activation of apoptosis in cells exposed to control siRNA; however, transfection with siRNA against survivin resulted in a marked activation of caspase 3 in A549 as well as a cleavage of their substrate poly (ADP-ribose) polymerase (PARP) (Figure 1D). In the case of transfected H520, where the silencing of survivin was less evident, the increase of the activated form of caspase 3 was more subtle, but also significant (Figure 1D). Cleaved PARP was also increased in H520 with silenced survivin. Finally, we wanted to elucidate whether the X-linked inhibitor of apoptosis protein (XIAP), a survivin partner in apoptosis inhibition, may also be affected after survivin silencing, since its stability against ubiquitin-dependent degradation is linked to survivin binding [10]. In our cellular models, XIAP also showed a significant reduction of its protein levels after 48 h of survivin downregulation, although less pronounced than survivin decrease, both in A549 and H520 cells (Figure 1D,E). Therefore, survivin appears as a potential therapeutic target in all lung cancer subtypes, suggesting the promising use of compounds targeting survivin for lung cancer treatment.

### 3.2. Indole-Based Tambjamine Analog 21 (T21) Downregulates Survivin in Lung Cancer In Vitro and In Vivo Models

**Figure 2:**
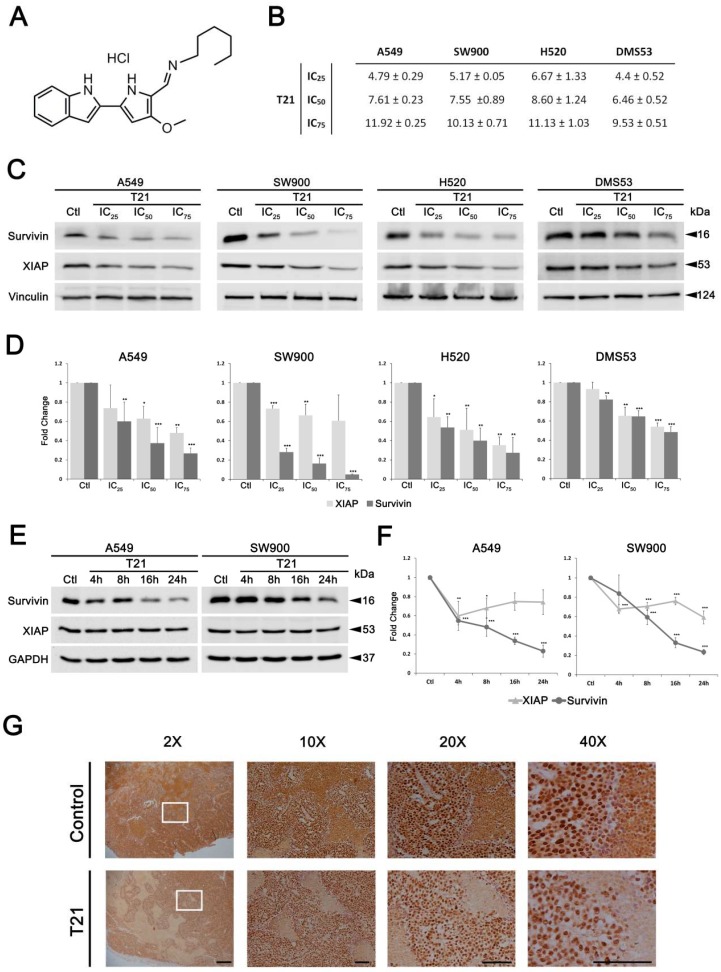
T21 decreases survivin levels in vitro and in vivo. (**A**) T21 chemical structure. (**B**) T21 inhibitory concentration (IC) values calculated in cell viability assays performed in several lung cancer cell lines. Some IC_50_ values were previously published in Manuel-Manresa et al. [6]. Values represent mean ± SD in µM. (**C**,**D**) After 24 h of treatment with IC_25_, IC_50_, IC_75_ values of T21 (Table S1), the expression of survivin and XIAP was analyzed by Western blot analysis in A549, SW900, H520 and DMS53 cell lines. (**E**,**F**) A549 and SW900 cells were treated with T21 at IC_50_ during different time periods (4, 8, 16, 24 h) followed by survivin and XIAP expression analysis through Western blot analysis. Results were obtained from at least three independent experiments. Bars represent the mean ± SD. Statistically significant results are indicated as *, *p*-value < 0.05; **, *p*-value < 0.01 and ***, *p*-value < 0.001. (**G**) Subcutaneous tumors from implanted DMS53 cells in Crl:NU-Foxn1nu mice were treated with 6 mg/kg of T21 every other day for 20 days. Tumor samples were stained against survivin by immunohistochemistry. Representative illustrations are shown at 2×, 10×, 20× and 40× magnifications, the scale bars correspond to 500, 500, 100 and 100 μm, respectively.

To extend the reported cytotoxic properties of T21 (Figure 2A), the effect on cell viability of H520 squamous cell lung cancer cells was also evaluated after treatment with different concentrations of T21 for 24 h, showing similar results than in the other studied cancer cells (Figure 2B). Moreover, the ability to survive and form colonies after T21 treatment was evaluated in a clonogenic assay in A549 cells (Figure S2). These cells were treated with T21 during 24 h, counted, and 200 viable cells were seeded in a new plate with fresh medium for one week. The survival capacity significantly decreased in cells after T21 treatment, especially in those treated with 10 µM, corroborating the potent cytotoxic effect of this compound.

In order to characterize in detail the molecular mechanism of action of this anticancer compound, we analyzed whether T21 was able to inhibit survivin in different lung cancer histological subtypes. For that purpose, the inhibitory effect of T21 on survivin levels was evaluated in non-small cell lung cancer SW900, H520 and A549 cells as well as in the small cell lung cancer DMS53 cells. All cells were treated with T21 at different cell viability inhibitory concentrations for 24 h (IC_25_, IC_50_ and IC_75_ concentrations) (Figure 2B). The results showed a sharp and significant dose-dependent decrease of survivin in all four cell lines (Figure 2C,D). Furthermore, T21 was also able to decrease, in a dose-dependent manner, the protein levels of XIAP in A549, H520 and to a lesser extent in SW900 and DMS53. Hence, these results suggest that the anti-apoptotic proteins survivin and XIAP decrease in a dose-dependent manner after T21 treatment allowing the induction of apoptosis.

To further investigate whether the effect of T21 over survivin and XIAP expression was simultaneous or not, SW900 and A549 were treated at their IC_50_ values for different time periods (4, 8, 16 and 24 h). The results showed a progressive and sharp decrease of survivin in a time-dependent manner in A549 and SW900, reaching a significant decrease at 24 h. Conversely, XIAP moderately decreased its protein levels at 4 h and were stabilized over time in both cell lines (Figure 2E,F). Hence, these results suggest that T21 potently inhibits survivin protein levels in a dose and time-dependent manner, whilst a moderate XIAP inhibition is observed.

On the other hand, we proposed to analyze the decrease of survivin levels after T21 treatment in subcutaneous tumors implanted in the flank of mice that were treated intraperitoneally with 6 mg/kg of T21 every other day for 20 days. As observed in Figure 2G, T21 was able to considerably reduce the protein levels of survivin in tumor samples from treated mice, which showed a decrease in tumor growth. This result corroborates the molecular changes induced by T21 in vitro showing that T21 decreases the expression of survivin also in tumors. Additionally, these results confirm that T21 has reached the tumors and suggest that survivin may be a good biomarker for T21 treatment efficacy.

### 3.3. T21 Reduces Survivin Levels via Gene Transcription Repression in Lung Cancer Cells

**Figure 3:**
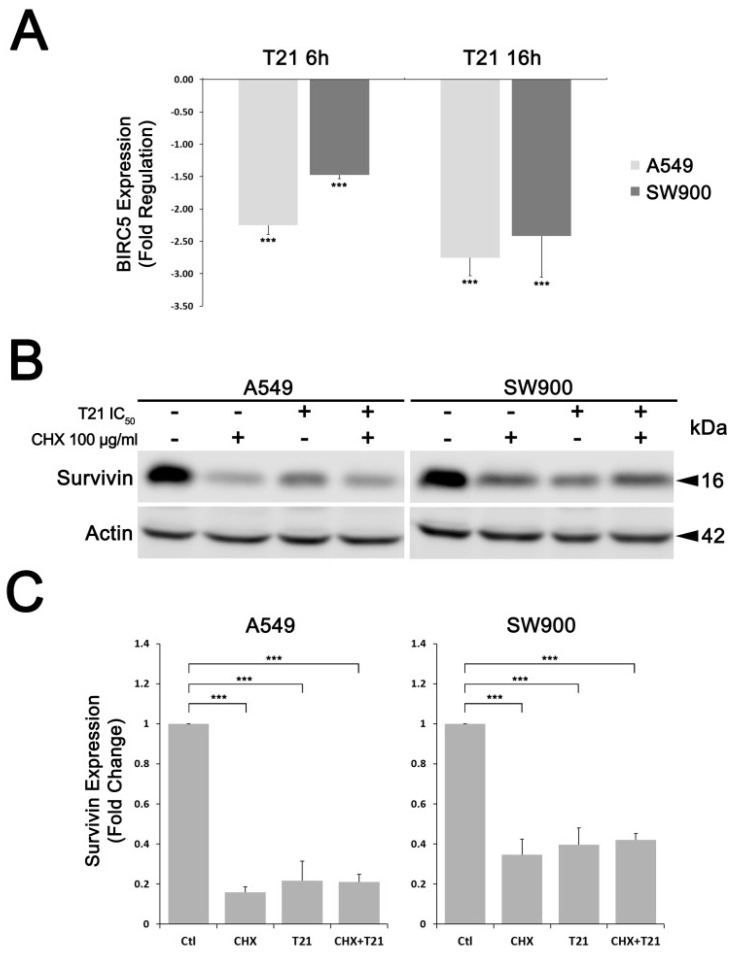
T21 reduces survivin levels through transcriptional repression. (**A**) A549 and SW900 cells were treated with T21 at IC_50_ for 6 and 16 h and BIRC5 gene expression was analyzed by RT-qPCR. (**B**,**C**) survivin levels were assessed by Western blot analysis in A549 and SW900 cells exposed to CHX at 100 µg/mL for 30 min followed by T21 treatment at IC_50_ during 24 h. Results were obtained from at least three independent experiments. Bars represent the mean ± SD. Statistically significant results are indicated as *, *p*-value < 0.05; **, *p*-value < 0.01 and ***, *p*-value < 0.001.

In order to identify the cellular mechanism that T21 was triggering to decrease survivin levels, A549 and SW900 cell lines were treated with T21 IC_50_ for 6 and 16 h and BIRC5 gene expression was analyzed by RT-qPCR. T21 was able to downregulate BIRC5 at both incubation periods albeit being more evident at 16 h, indicating that T21 represses survivin gene expression (Figure 3A).

To further study whether the reduction of survivin levels observed after T21 exposure was only due to a transcriptional repression, SW900 and A549 were treated with CHX, an inhibitor of protein synthesis. After 30 min of CHX treatment, cells were treated with T21 at IC_50_ for 24 h. As observed in Figure 3B,C, survivin levels in both cell lines treated with T21 plus CHX were similar to CHX treated cells. Hence, T21 was inducing the same effect that only inhibiting the de novo protein synthesis, since no additional degradation was observed compared to CHX treated cells. Therefore, these results suggest that the reduction of survivin levels after T21 exposure is mainly due to a transcriptional repression and T21 is not triggering direct protein degradation.

### 3.4. T21 Suppresses STAT3 Phosphorylation via JAK/STAT3 Pathway Inhibition in Lung Cancer Cells

**Figure 4:**
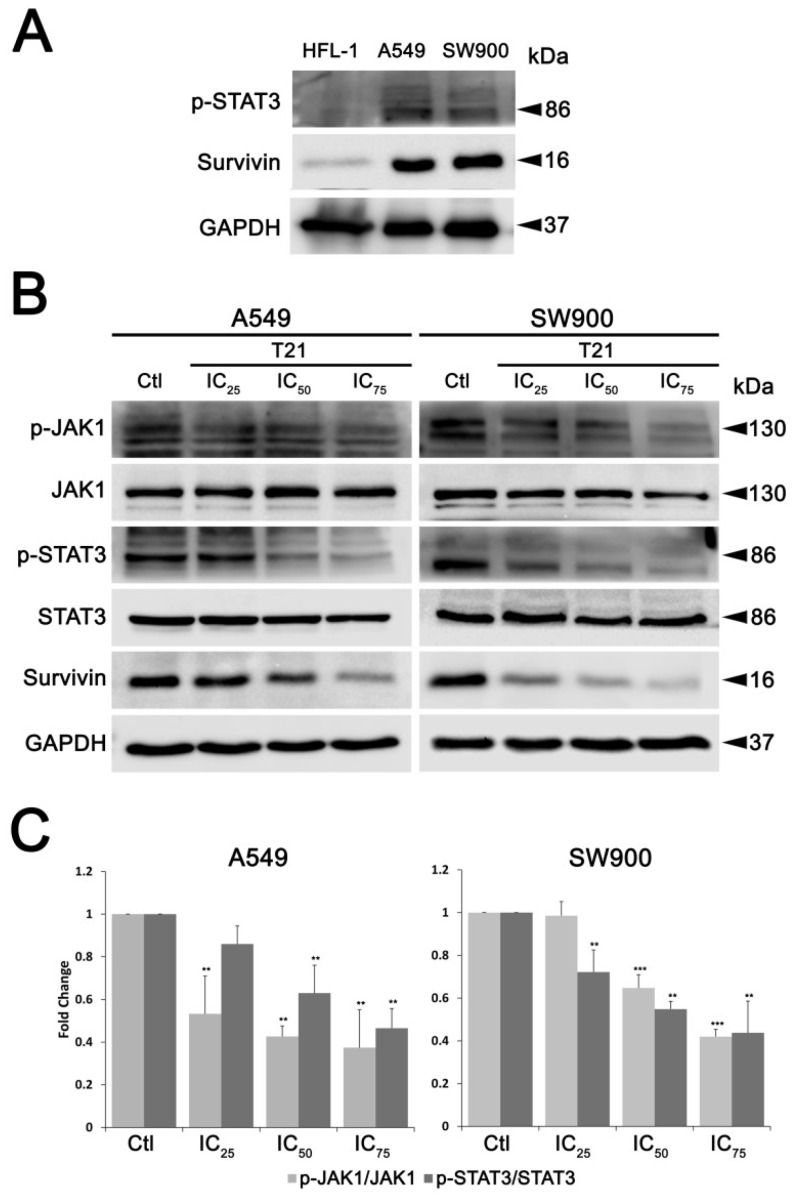
STAT3 pathway analysis. (**A**) STAT3 signaling in lung normal and cancer cells. Levels of phospho-STAT3 and survivin were analyzed by western blot in HFL-1, A549 and SW900 cells. (**B**,**C**) JAK/STAT3 pathway inactivation after T21 treatment. After 24 h of treatment with the IC_25_, IC_50_, IC_75_ values of T21 (Table S1), the expression and phosphorylation levels of several members of JAK1/STAT3 pathway were analyzed by Western blot in A549 and SW900 cells. Results were obtained from at least three independent experiments. Bars represent the mean ± SD. Statistically significant results are indicated as *, *p*-value < 0.05; **, *p*-value < 0.01 and ***, *p*-value < 0.001.

Among other signaling pathways, the JAK/STAT3 has been described to be involved in increasing survivin expression levels and promoting tumorigenesis after aberrant activation in cancer cells [11]. In this regard, we first evaluated STAT3 activity in A549 and SW900 lung cancer cells, compared to normal cells (HFL-1) (Figure 4A), observing a significant increase in basal STAT3 signaling in cancer cells, accompanied by higher levels of survivin expression.

Then, in order to assess whether this pathway could be involved in survivin regulation after T21 treatment, STAT3 phosphorylation at Tyr705 (p-STAT Y705) was evaluated in SW900 and A549 cells after T21 exposure at their IC_25_, IC_50_ and IC_75_ for 24 h. This phosphorylation allows STAT3 dimerization, nucleus translocation from cytoplasm, and DNA binding [12]. Both cell lines showed a significant reduction in net phosphorylation of STAT3 at Tyr705 after T21 treatment in a dose-dependent manner (Figure 4B,C), similarly to that observed in survivin levels. Since STAT3 can be phosphorylated by JAK, upon IL-6 stimulation and gp-130 receptor activation, phosphorylation status of this upstream STAT3 activator was also evaluated. JAK1 showed a significant reduction on its phosphorylation state at Tyr1034/1035 (p-JAK1 Y1034/1035). Similarly, JAK2 phosphorilation decreased after T21 treatment (Figure S3). These results suggest that T21 inhibits STAT3 phosphorylation through JAK/STAT3 pathway, which may reduce the gene expression of survivin provoking the observed protein level decrease.

### 3.5. T21 Blocks IL-6-Induced STAT3 Phosphorylation in A549

**Figure 5:**
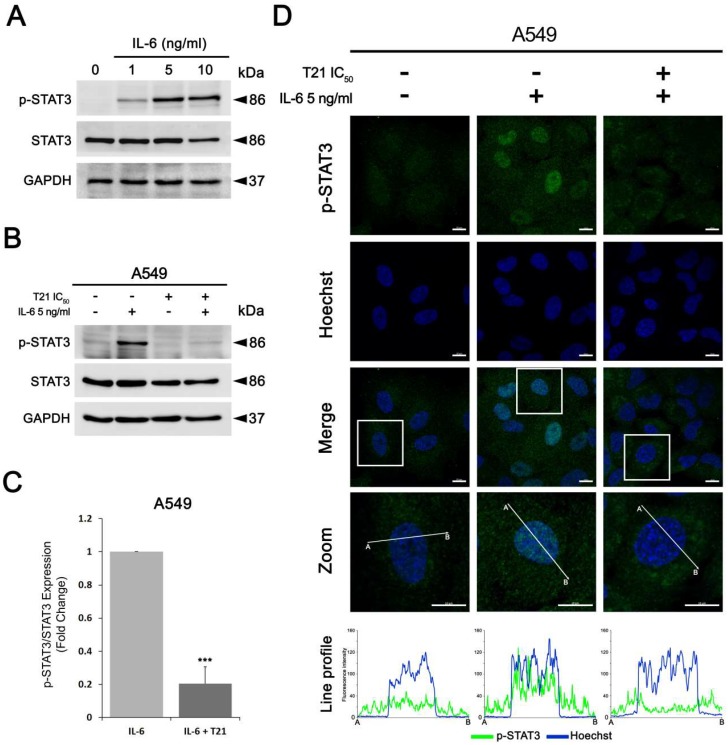
T21 blocks STAT3 activity after IL-6 stimulation. (**A**) A549 cells were starved, then treated with different concentrations of IL-6 and phosphorylation levels of STAT3 were assessed by Western blot analysis. (**B**,**C**) after starvation, A549 were pretreated with IC_50_ T21 for 4 h followed by 5 ng/mL of IL-6 stimulation for 30 min. Phosphorylation levels of STAT3 were then assessed by Western blot analysis. Bars represent the mean ± SD. Statistically significant results are indicated as *, *p*-value < 0.05; **, *p*-value < 0.01 and ***, *p*-value < 0.001. (**D**) A549 cells were pretreated with IC_50_ T21 for 4 h, after starvation, followed by 5 ng/mL of IL-6 stimulation for 30 min. Subcellular localization of phospho-STAT3 (Y705) was assessed by immunofluorescence staining. The framed regions are zoomed in the bottom images. The line profiles of phospho-STAT3 (Y705) and Hoechst signals were measured by ZEN 2.3 blue edition (Carl Zeiss) software. (Scale bars correspond to 10 µm).

To examine whether T21 was also able to block STAT3 signaling after stimulating with one of its upstream ligands, we first analyzed the IL-6 effects on STAT3 phosphorylation in lung cancer cells. A549 cells, after starvation for at least 12 h to decrease the JAK/STAT signaling pathway, were stimulated with IL-6 at different concentrations for 30 min. STAT3 was activated by phosphorylation in a dose-dependent manner after IL-6 stimulation from 1 to 10 ng/mL (Figure 5A). In turn, the total STAT3 expression level was not altered with IL-6 stimulation. Next, in the same culture conditions after starvation, A549 cells were pretreated with T21 at IC_50_ for 4 h followed by 5 ng/mL of IL-6 stimulation for 30 min. The results showed that IL-6 treatment induced STAT3 phosphorylation, but this induction was almost totally repressed by T21 in pretreated cells, showing that T21 pretreatment impedes activation of this signaling pathway by IL-6 (Figure 5B,C).

Additionally, to investigate whether T21 can block the IL-6-induced translocation of STAT3 from cytoplasm to nucleus, A549 cells were pretreated with T21 at IC_50_ for 4 h followed by 5 ng/mL of IL-6 stimulation for 30 min. After cell fixation and immunofluorescence staining, phosphorylated STAT3 at Tyr705 was increased and translocated into nucleus due to IL-6 stimulation (Figure 5D). By contrary, cells stimulated with IL-6 after T21 treatment showed lower levels of phosphorylated STAT3 as well as no significant translocation into the nucleus. Altogether, the above results suggest that T21 is able to prevent IL-6-induced STAT3 phosphorylation and its nuclear translocation; hence T21 blocks the JAK/STAT3 signaling pathway in lung cancer cells.

## 4. Discussion

Although there have been significant advances in lung cancer management in recent years, lung cancer overall survival remains very low [13]. Hence, more efforts are needed to identify, design and develop new compounds aimed at treating lung cancer. The development of new technologies are aiding to find promising lead candidates from natural products, which have demonstrated to be a major source for drug discovery along history [14]. Natural products and their bioactive derivatives from animals, plants, fungi, and microorganisms, among others, have widely been studied for therapeutic use, being the morphine the first commercial plant-derived product in 1826. Interestingly, about a quarter of all Food and Drug Administration (FDA) and/or the European Medical Agency (EMA) approved drugs are directly or indirectly plant based [15]. Natural-based products have also been an important source of several clinically useful anti-cancer agents, as the well-known antineoplastic paclitaxel that derives from endophytic fungi isolated from plants and is used for the treatment of breast, ovarian and lung cancer. In this regard, we have recently described a marine organism-derived small molecule called indole-based tambjamine analog 21 (T21), which possesses a potent antitumor effect through the induction of apoptosis [6]. In accordance with our findings, several studies also demonstrated the potential of compounds derived from marine organism as anti-cancer agents, being some of these compounds in clinical trials and others already approved for clinical use [4]. Various examples of approved antineoplastic analogs derived from marine organisms include cytarabine (Cytosar), an antimetabolite drug used for the treatment of various types of leukemia; trabectedin (Yondelis), a DNA alkylator for soft sarcoma treatment; and the antimitotic compounds that inhibits microtubule dynamics eribulin mesylate (Halaven) and brentuximab vedotin (Acentris), which are used in breast cancer and Hodgkin’s lymphoma treatment, respectively. Regarding the cell death induced by T21, this pro-apoptotic compound significantly decreases survivin levels, also in in vivo studies, inducing simultaneously a significant decrease in tumor volume without any obvious toxicity in mice [6]. As a member of the inhibitor of apoptosis protein (IAP) family, survivin (protein encoded by BIRC5) plays an important role in tumorigenesis, metastasis and therapy resistance [16,17]. Survivin is highly expressed during embryonic and fetal development, but is almost undetectable in most normal finally differentiated tissues [8]. In cancer cells, survivin is overexpressed, being associated with poor prognosis in many human neoplasms. Moreover, survivin also participates in complex molecular signaling cascades cancer-related, being therefore crucial for carcinogenesis [18]. Altogether, the fact that survivin is overexpressed in tumors as well as its key biological roles that promote carcinogenesis and chemoresistance, makes survivin a promising therapeutic target to treat cancer [8]. In these sense, putative survivin antagonists under study are showing promising antitumoral potential, such as YM155, a small-molecule inhibitor that targets and suppresses specifically the activity of the survivin promoter [19]. Nevertheless, despite showing good clinical results in combination regimens, YM155 and other survivin inhibitors under study present modest activity as a single agent, which may be attributed to incomplete or transient survivin inhibition.

In order to characterize in depth the molecular mechanism by which this compound inhibits survivin, we analyzed its effects on several lung cancer cells. Here, we show that T21 significantly reduced the levels of survivin in all survivin overexpressing cells. Interestingly, T21 also induced a decrease of survivin levels in vivo, suggesting an acceptable metabolism rate limiting biotransformation of this drug before exerting its effects. On the other hand, T21 was also able to reduce the levels of XIAP, another IAP protein which interacts and forms a complex with survivin to inhibit the effector caspases [20]. In this context, it has also been reported that this IAP-IAP complex enhances XIAP stability against ubiquitin-dependent degradation [10]. Hence, T21 effect over survivin expression and protein levels might be affecting the stability of XIAP, thus avoiding caspase inhibition and finally promoting apoptosis. Furthermore, we showed how the effect of T21 on survivin was not due to direct protein degradation but survivin gene repression. These results clearly categorize T21 as an indirect inhibitor of survivin, like herceptin, lapatinib or SD-1029, among others, which downregulates survivin expression targeting key cellular signaling pathways involved in the expression of this protein [19,21,22,23].

In this regard, survivin gene regulation can be triggered by several signaling pathways, such as PI3K/AKT, MAPK/ERK and JAK/STAT pathways [7]. Among them, STAT3 has been described as an important signaling mediator in malignant diseases by promoting the expression of genes involved in cancer proliferation, cell survival, immune suppression, inflammation, and metastasis [24,25]. Persistent activation of STAT3, which is found in 22–65% of NSCLC [26], induces survivin expression and thereby prevents apoptosis, which potentially contributes to resistance to chemotherapy [11]. In this study, we found that T21 treatment could inhibit the STAT3 Tyr705 phosphorylation in NSCLC cells. Interestingly, it has been proved that the acidification of the cytosol in A549 cells triggers a rapid dephosphorylation of STAT3 at Tyr705 [27]. In this regard, T21 significantly decreases the intracellular pH in A549 cells after 1 h, promoting the acidification of the cytosol [28]. Hence, the rapid dephosphorylation of STAT3 at Tyr705 observed in our A549 cells may be a consequence of this cytosolic acidification produced by T21. Moreover, since the transcriptional activity of STAT3 is closely associated with this phosphorylation, the inhibition of the JAK/STAT3 pathway may be the cause of the observed downregulation of survivin in our model. In fact, the repression of STAT3 transcriptional activity after T21 treatment is also supported by our previous results, where other STAT3 downstream genes, such as Bcl-2, Bcl-xL or Mcl-1 were also modified after treatment [6]. Furthermore, our results are consistent with previous studies that already described a reduction of survivin in glioblastoma, leukemia, NSCLC, breast and ovary cancers when JAK/STAT3 pathway was inhibited [23,29,30,31,32].

On the other hand, STAT3 activation is commonly triggered by the binding of growth factors and cytokines to their specific receptors. IL-6, a pleiotropic cytokine, has been associated with STAT3 activation after its binding to the gp130 receptor, which in turn, recruits and triggers the activation of JAK [33]. JAK can then phosphorylate STAT3 on Try705 residue, promoting its homodimerization and translocation to the nucleus, followed by gene expression regulation [34]. Data from previous studies have confirmed that elevated IL-6 levels are observed in patients with a variety of cancers [35]. In particular, IL-6 levels have been found increased in nearly 40% of lung cancer patients [36]. Furthermore, high levels of circulating IL-6 were associated with poor responses and worst survival outcomes in NSCLC patients treated with chemotherapy [37]. In this study, we confirmed that IL-6 was able to phosphorylate STAT3 at Tyr705 as well as promote its nuclear translocation. Conversely, both activation and nuclear translocation of STAT3 were repressed after T21 treatment, suggesting that T21 may help to overcome the chemoresistance of tumors with high IL-6 levels.

## 5. Conclusions

We have investigated in detail the molecular mechanisms of action by which the novel anticancer drug T21 inhibits the antiapoptotic protein survivin in vitro as well as in vivo and have validated this protein as a promising therapeutic target for lung cancer treatment. As a result, we have demonstrated that JAK/STAT3 signaling pathway is the molecular mechanism involved in the inhibition of survivin by T21. T21 regulates survivin by transcriptional gene repression, blocking the phosphorylation of STAT3 and its nuclear translocation. Moreover we have shown that STAT3 inhibition by T21 is effective even in the presence of IL-6 overstimulation. These findings reinforce the potential of T21 as a novel chemotherapeutic agent for lung cancer treatment, especially for lung cancer patients with high levels of IL-6, who show poor outcomes in the clinics. Altogether, T21 is a novel potent inhibitor of STAT3 transcription factor that significantly decreases survivin levels, which may sensitize cancer cells to apoptosis and may enhance the apoptotic effect induced by traditional chemotherapeutic drugs in lung cancer patients.

## Supplementary Materials

The following are available online at https://www.mdpi.com/2218-273X/9/8/361/s1, Figure S1: Survivin overexpression validation by immunofluorescence in HFL-1, A549 and SW900 cells. Figure S2: Effects of T21 on cell viability by a clonogenic assay. Figure S3: JAK2 activity after T21 treatment.
