# Supplementary material for: The Natural-Based Antitumor Compound T21 Decreases Survivin Levels through Potent STAT3 Inhibition in Lung Cancer Models
Source: Biomolecules. 2019 Aug 13;9(8):361. doi: 10.3390/biom9080361 (PMC6724027; doi:10.3390/biom9080361)
Supplement: Supplementary file 1 [file biomolecules-09-00361-s001.pdf]

biomolecules

MDPI

Supplementary Figures

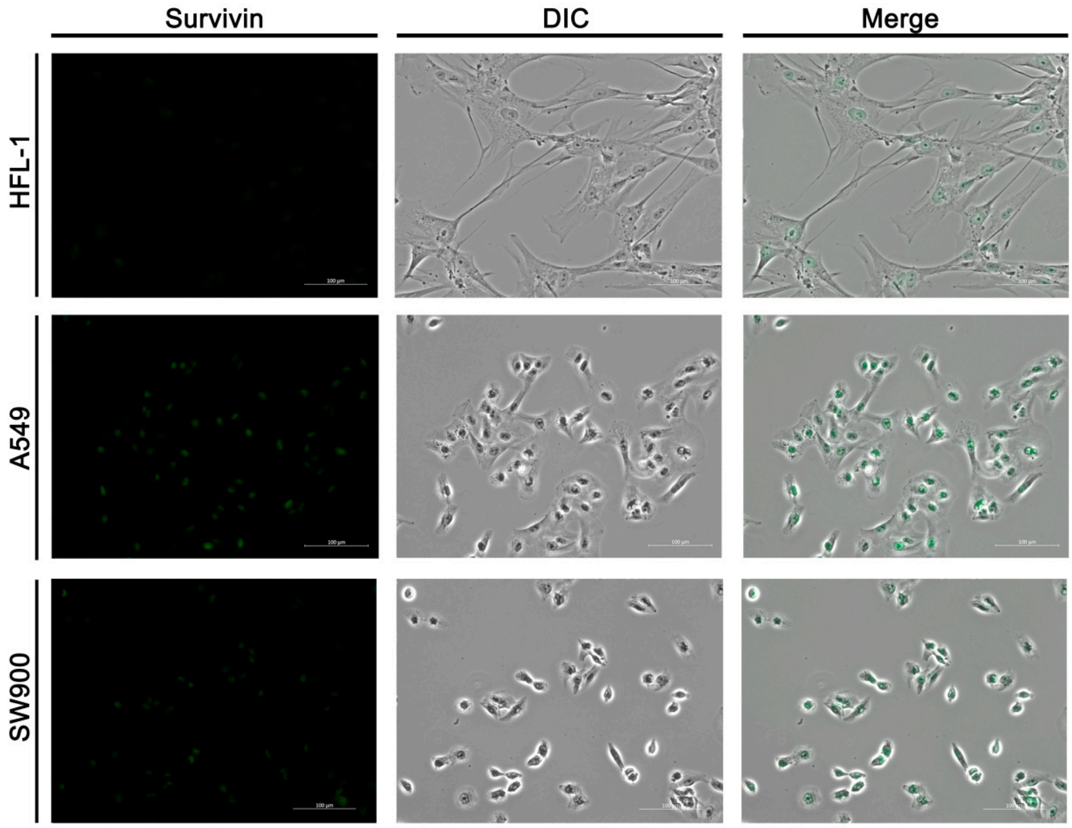

Figure S1. Survivin expression on normal (HFL-1) and lung cancer cells (A549, SW900). Survivin protein levels and localization were analyzed by immunocytochemistry.

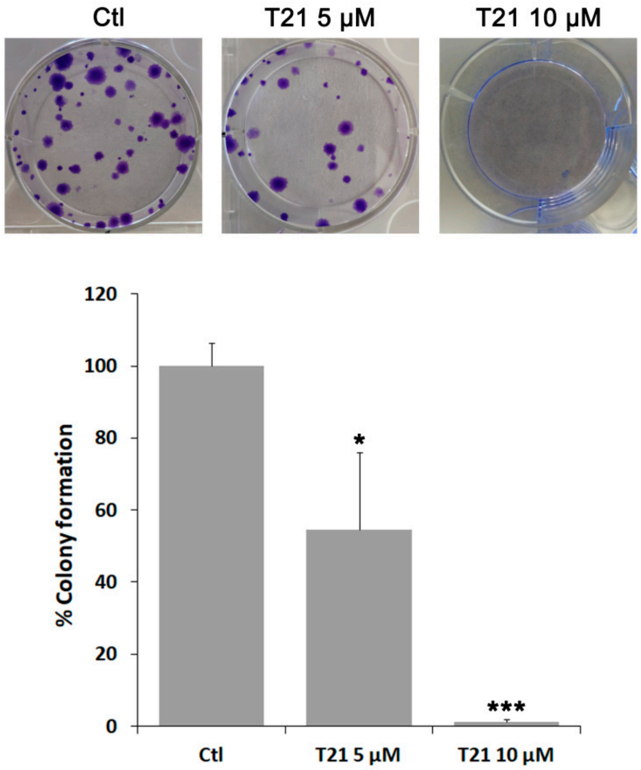

Figure S2. Effects of T21 on cell viability by a clonogenic assay. A549 cells were treated with T21 at concentrations ranging 2.5 – 10 μM for 24 h. A clonogenic assay was performed, (**A**) colonies were counted and (**B**) percentage of colony formation was calculated referred to control cells.

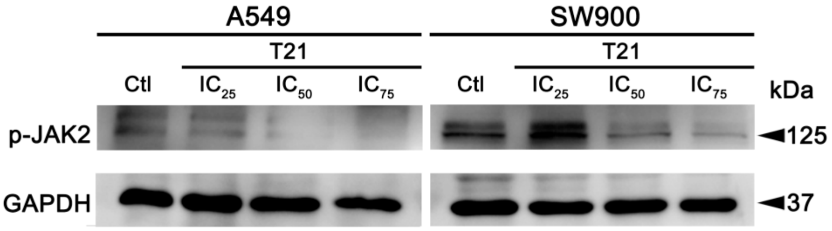

Figure S3. JAK2 activity after T21 treatment. JAK2 phosphorylation levels were assessed by immunoblot after treating A549 and SW900 cells with their respective IC values for 24 h.
